# Supplementary material for: Pseudomonas Exotoxin A Based Toxins Targeting Epidermal Growth Factor Receptor for the Treatment of Prostate Cancer
Source: Toxins (Basel). 2020 Nov 28;12(12):753. doi: 10.3390/toxins12120753 (PMC7761469; doi:10.3390/toxins12120753)
Supplement: Supplementary file 1 [file toxins-12-00753-s001.pdf]

Supplementary Materials: Pseudomonas Exotoxin A Based Toxins Targeting Epidermal Growth Factor Receptor for the Treatment of Prostate Cancer

Alexandra Fischer, Isis Wolf, Hendrik Fuchs, Anie Priscilla Masilamani and Philipp Wolf

Table S1. Binding of EGF and the targeted toxins EGF-PE40 and EGF-PE24mut on PC cells. The Equilibrium dissociation constants (K_D) were defined as half-maximal saturation concentrations determined by flow cytometry. nd, not determinable.

Title	LNCaP	DU145	PC-3	CHO
	K_D(nM)	K_D(nM)	K_D(nM)	K_D(nM)
EGF	3.8	3.1	4.4	nd
EGF-PE40	108.8	235.9	244.2	nd
EGF-PE24mut	35.0	36.9	26.6	nd

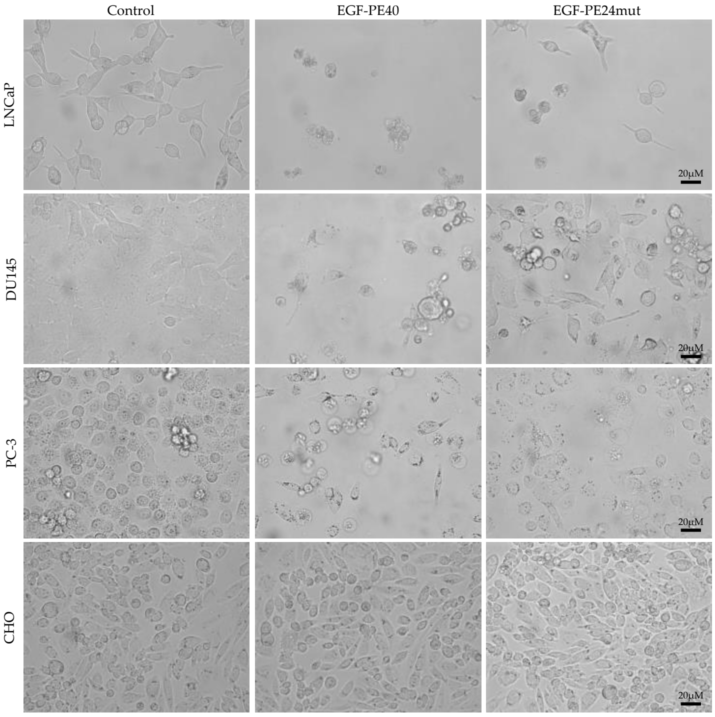

Figure S1. Morphological changes of PCa cells upon treatment with EGF-based targeted toxins. Microphotographs of EGFR-positive LNCaP cells treated with 0.7 nM targeted toxins for 48h, DU145 cells treated with 1 nM targeted toxins for 72 h, PC-3 cells treated with 2 nM targeted toxins for 72 h, and EGFR-negative CHO cells treated with 2 nM targeted toxins for 72 h. The scale bar represents 20 µM.

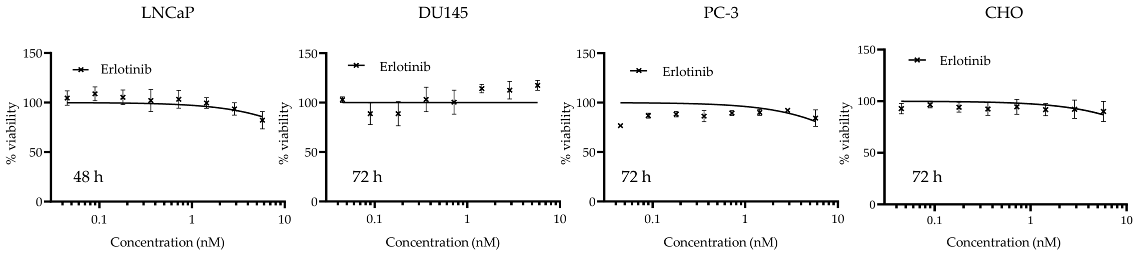

Figure S2. Influence of the EGFR inhibitor erlotinib on the viability of PCa cells. EGFR-positive LNCaP, DU145, PC-3 and EGFR-negative CHO cells were incubated with the EGFR inhibitor erlotinib for the indicated periods of time. Reduction of cell viability was analysed by WST-1 assay. Mean values ± SEM of three independent experiments.

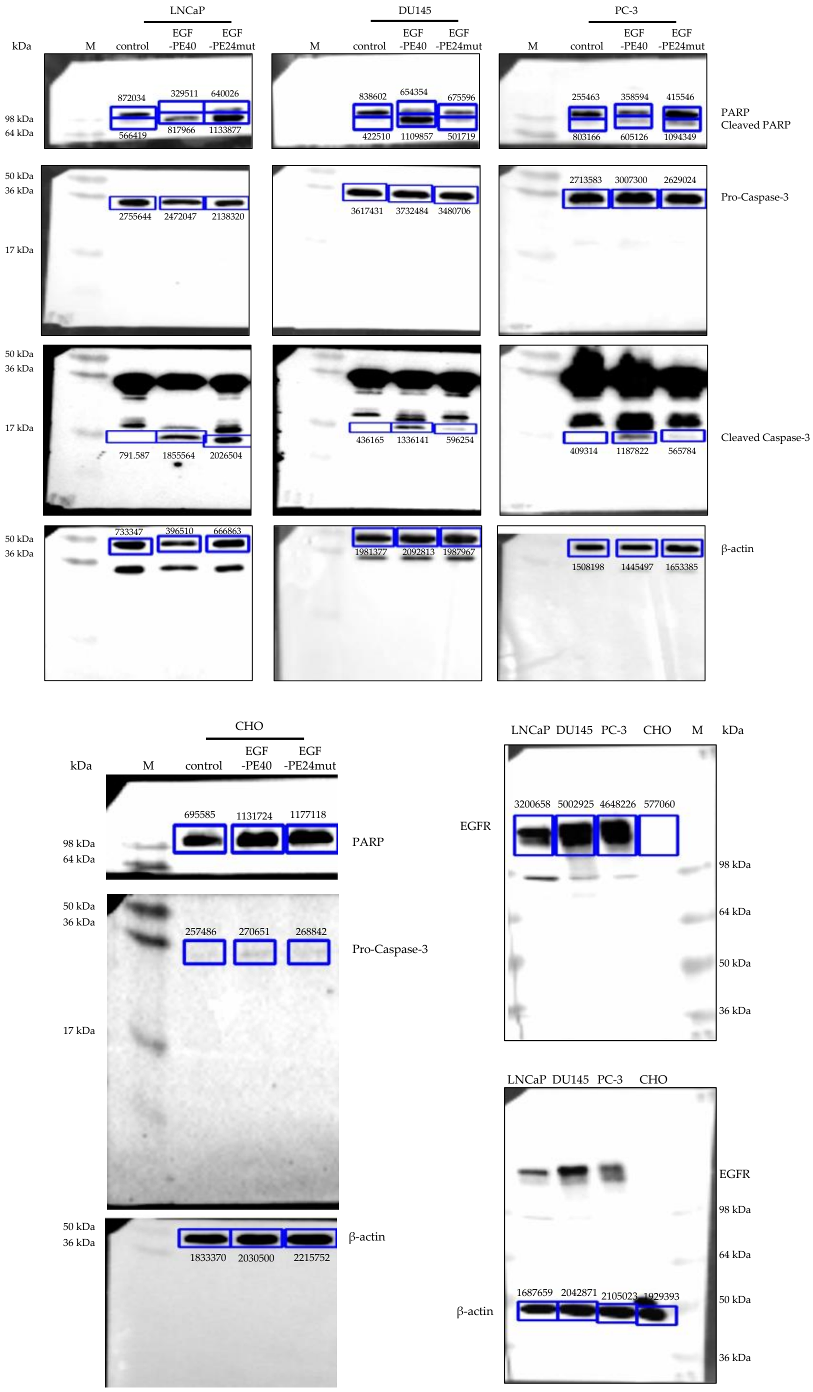

Figure S3. Whole Western Blots including densitometry ratios.
